# Strawberries Improve Pain and Inflammation in Obese Adults with Radiographic Evidence of Knee Osteoarthritis

**DOI:** 10.3390/nu9090949

**Published:** 2017-08-28

**Authors:** Jace Schell, R. Hal Scofield, James R. Barrett, Biji T. Kurien, Nancy Betts, Timothy J. Lyons, Yan Daniel Zhao, Arpita Basu

**Affiliations:** 1 Nutritional Sciences, Oklahoma State University, Stillwater, OK 74078, USA; jace.schell@okstate.edu (J.S.); nancy.betts@okstate.edu (N.B.); 2 Section of Endocrinology and Diabetes, University of Oklahoma Health Sciences Center, Oklahoma City, OK 73104, USA; Hal-Scofield@omrf.org (R.H.S.); biji-kurien@omrf.org (B.T.K.); 3 Arthritis and Clinical Immunology, Oklahoma Medical Research Foundation, Oklahoma City, OK 73104, USA; 4 Medical Service, US Department of Veterans Affairs Medical Center, Oklahoma City, OK 73102, USA; 5 Department of Family and Preventive Medicine, University of Oklahoma Health Sciences Center, Oklahoma City, OK 73104, USA; jim-barrett@ouhsc.edu; 6 Division of Endocrinology, Medical University of South Carolina, Charleston, SC 29425, USA; lyonstj@musc.edu; 7 Biostatistics and Epidemiology, University of Oklahoma Health Sciences Center, Oklahoma City, OK 73104, USA; Daniel-zhao@ouhsc.edu; 8 Kinesiology and Nutrition Sciences, University of Nevada Las Vegas, Las Vegas, NV 89154, USA

**Keywords:** strawberries, knee osteoarthritis, pain, inflammation

## Abstract

Osteoarthritis (OA), the most common form of arthritis, is a significant public health burden in U.S. adults. Among its many risk factors, obesity is a key player, causing inflammation, pain, impaired joint function, and reduced quality of life. Dietary polyphenols and other bioactive compounds in berries, curcumin, and tea have shown effects in ameliorating pain and inflammation in OA, but few clinical studies have been reported. The purpose of the present study was to examine the effects of dietary strawberries on pain, markers of inflammation, and quality of life indicators in obese adults with OA of the knee. In a randomized, double-blind cross-over trial, adults with radiographic evidence of knee OA (*n* = 17; body mass index (BMI): (mean ± SD) 39.1 ± 1.5; age (years): 57 ± 7) were randomized to a reconstituted freeze-dried strawberry beverage (50 g/day) or control beverage daily, each for 12 weeks, separated by a 2-week washout phase (total duration, 26 weeks). Blood draws and assessments of pain and quality of life indicators were conducted using the Visual Analog Scale for Pain (VAS Pain), Measures of Intermittent and Constant Osteoarthritis Pain (ICOAP), and Health Assessment Questionnaire-Disability Index (HAQ-DI) questionnaires, which were completed at baseline and at weeks 12, 14, and 26 of the study. Among the serum biomarkers of inflammation and cartilage degradation, interleukin (IL)-6, IL-1β, and matrix metalloproteinase (MMP)-3 were significantly decreased after strawberry vs. control treatment (all *p* < 0.05). Strawberry supplementation also significantly reduced constant, intermittent, and total pain as evaluated by the ICOAP questionnaire as well as the HAQ-DI scores (all *p* < 0.05). No effects of treatment were noted on serum C-reactive protein (CRP), nitrite, glucose, and lipid profiles. Dietary strawberries may have significant analgesic and anti-inflammatory effects in obese adults with established knee OA.

## 1. Introduction

Osteoarthritis (OA), the most common type of arthritis, is a chronic, painful, and inflammatory musculoskeletal disease causing functional impairment in approximately 27 million Americans; obesity and advancing age are important risk factors [1,2]. While there is no cure, the current management of OA combines nonpharmacological and pharmacological interventions, and often involves costly joint replacement procedures [3]. Non-steroidal anti-inflammatory drugs may lead to gastrointestinal side-effects, and effective, safer alternatives could benefit millions of patients. Nutraceuticals are good candidates for the management of OA, due to their safety profile and potential efficacy. However, the popularly used supplements, such as glucosamine, chondroitin sulfate, and avocado–soy unsaponifiables, have failed to show a convincing and significant mitigation of symptoms in a meta-analysis of randomized clinical trials, or lack long-term studies on the clinical symptoms and biomarkers of knee OA [4,5,6].

Obesity is considered a significant risk factor for OA, and contributes to the chronic inflammation that underlies the pathogenesis and symptoms of the condition [7,8]. Biomarkers of inflammation, especially serum C-reactive protein (CRP) and interleukin-6 (IL-6), and those of cartilage degradation, including matrix metalloproteinases (MMPs), have been positively correlated with pain and the progression of OA [9,10,11,12]. Dietary bioactive compounds, such as curcumin [13,14], ginger [15], green tea polyphenols [16], and herbal tea [17] have been shown to be effective in the management of pain symptoms and in reducing inflammatory biomarkers of OA. However, such clinical studies are few in number and of short duration, mostly examine pain symptoms but not disease biomarkers, and involve non-obese and otherwise healthy participants. Among the nutritional supplements and foods containing bioactive compounds, polyphenol-rich dietary berries have been extensively studied for their protective associations with other chronic conditions, including hypertension [18], type 2 diabetes [19], and overall inflammation [20], as reported in epidemiological studies. Berries, such as blueberries and red raspberries, have been shown to reduce the symptoms and progression of arthritis, such as pain and articular degeneration, in collagen-induced experimental models of arthritis [21,22]. Our group has previously reported the safety and efficacy of berries, especially freeze-dried blueberries and strawberries, in improving features of the metabolic syndrome and decreasing surrogate biomarkers of atherosclerosis in clinical studies [23,24,25]. To our knowledge, no previous clinical study has been reported on the effects of berries on OA of the knee in adults.

For this reason, we undertook the present study to examine the effects of freeze-dried strawberries on pain symptoms and on circulating biomarkers of inflammation and cartilage degradation in obese adults with symptomatic knee OA. Our primary aim was to determine the effects of freeze-dried strawberries on pain scores assessed by the Visual Analog Scale for Pain (VAS Pain) and those based on a Measure of Intermittent and Constant Osteoarthritis Pain (ICOAP) survey, as well as on selected biomarkers of inflammation and cartilage degradation associated with knee OA in comparison to a control group.

## 2. Materials and Methods

### 2.1. Participants

Obese participants with a body mass index (BMI) >30 kg/m^2^, a large waist circumference (>35 in for women and >40 in for men), and radiographic evidence of knee OA were enrolled in the study. A diagnosis of knee OA was verified by a rheumatologist based on the radiological evidence of mild to moderate bilateral primary knee OA defined by the American College of Rheumatology (ACR) [26]. Radiological evidence of degenerative OA, but without fractures or dislocation, confirmed eligibility for the study. Participants were excluded if they had any of the following conditions: previous knee surgery, rheumatoid arthritis, a metabolic disorder (such as diabetes and cancer), liver or kidney failure, pregnant or lactating, use of corticosteroids and/or intra-articular injections during the preceding 3 months, use of fish oils and glucosamine, participation in a weight loss program in the preceding 6 months, and recent changes in physical activity levels, regular smoking, or allergic to strawberries. In addition, participants who were unable to express their pain (such as those with any mental condition) were also excluded from the study. The study (the ethics approval code: HE1517) was approved by the ethics committees at the University of Oklahoma Health Sciences Center (OUHSC) and at Oklahoma State University (OSU). All participants provided written informed consent prior to enrollment in the study. The trial was registered with clinicaltrials.gov (NCT02518347).

### 2.2. Study Design and Intervention

Participants were recruited at the Oklahoma Clinical and Translational Sciences Institute (OCTSI) at OUHSC and at the Department of Nutritional Sciences Clinical Assessment Unit at OSU. The recruitment was conducted though campus-wide e-mail advertisements and physician referrals. Upon qualification, the participants were randomly assigned to one of the two study groups in a 26-week crossover study: strawberry and control. Randomization was performed using a sequence of randomly generated numbers using SAS (Version 9.4; SAS Institute Inc., Cary, NC, USA). Each intervention was for 12 weeks, with and intervening two weeks of washout phase. During the active treatment phase, the participants consumed 50 g of freeze-dried strawberry powder reconstituted in water twice a day. This dose of strawberry powder is equivalent to approximately 500 g of fresh strawberries, and was previously used in another study [24]. The control powder was formulated to match the sensory properties of the strawberry powder as well as its caloric value and macronutrient composition. Table 1 shows the nutritional composition of the strawberry and control powders provided by the California Strawberry Commission (Watsonville, CA, USA). The nutrient and phytochemical composition of the strawberry and control powders was determined at the Robert M. Kerr Food and Agricultural Products Center at Oklahoma State University (Stillwater, OK, USA), and at the Brunswick Laboratories (Southborough, MA, USA), respectively. The participants were asked to consume the strawberry or control beverage twice a day, at similar time points that were at least six to eight hours apart, and also to consume the beverage as a snack by itself, and not with a meal or other snacks to prevent the confounding effects of other dietary factors. The participants were instructed to take the last dose of the test beverage at least 10–12 h prior to the fasting blood draw the following morning. The participants were also asked to refrain from consuming other berry products during the study, and to maintain usual diet and physical activity. Compliance was assessed by the return of unused test agents and a mandatory three visits per week to the clinic for supervised consumption and a determination of plasma ellagic acid [27].

### 2.3. Biochemical Variables

Freshly drawn blood samples were sent to the OU Medical Center laboratory for an analysis of a comprehensive metabolic panel, including serum glucose, lipid profiles, HbA1c, and high-sensitivity C-reactive protein (hs-CRP), using an automated clinical analyzer (Abbott Architect Instruments). Serum IL-6, IL-1β, and MMP-3 and 8 were measured using ELISA kits based on the manufacturer’s protocol (R&D Systems, Minneapolis, MN, USA) with inter-assay CVs (Coefficient of variation) of 4.5%, 7.6%, 3.6%, and 7.5%, respectively. Serum nitrite was measured using the Griess Reagent System (Promega Corporation, Madison, WI, USA) with a mean inter-assay CV of 3.3%.

### 2.4. Pain Scores and Quality of Life Indicators

Knee pain scores were assessed using the ICOAP survey, a multidimensional, OA-specific measure designed to provide a comprehensive evaluation of pain experience in people with knee OA, that has been used in several large studies [28,29]. The ICOAP is an 11-item scale evaluating two pain domains: a 5-item scale evaluating constant pain and a 6-item scale evaluating intermittent pain. We also used the VAS Pain and health scale to assess the visual perception of the participants’ pain intensity and feeling of well-being [30]. In addition, the Health Assessment Questionnaire-Disability Index (HAQ-DI) was used to assess functional ability using 20 items distributed across eight dimensions (dressing, arising, eating, walking, reach, grip, hygiene, and daily activity), rating each according to a four-level disability scale (range 0–3) [30]. The participants were asked to fill out the questionnaires on the morning of their fasting blood draws visits, at least 10–12 h following their last test dose of strawberry and control beverage.

### 2.5. Dietary Analysis

Habitual intake of food and beverages was recorded using 3-day food records (two weekdays and one weekend day) at baseline, and at week 6, 12, 14, 20, and 26 of the study. At enrollment, the participants were educated to record food and beverages by a study Registered Dietitian (RD) using food models and utensils for the estimation of portion sizes. Nutrient intakes were analyzed using Nutritionist Pro version 3.2 (Axxya Systems LLC, Redmond, WA, USA). The averages of three days were used to estimate the nutrient intakes per week for each participant.

### 2.6. Statistical Analysis

For baseline demographics and characteristics, continuous variables were expressed as means ± SD and discrete variables were presented as counts and proportions. Our main objective was to assess whether the selected biomarkers of inflammation and cartilage degradation, as well as knee pain scores, were different between the strawberry and control phases at 12 vs. 26 weeks of the crossover study. To test this aim, we used a linear mixed-effects model (PROC MIXED) with time as within-subject factor and intervention group as a between-subject factor for each variable. Data were corrected for baseline values. We also examined associations of the serum biomarkers with knee pain scores at baseline using a multiple linear regression model, adjusting for baseline age, BMI, and energy intake. The assumptions used in the sample size calculation were conservative, based on the report by Panahi et al. [13]. From previous dietary intervention studies in knee OA, we expected a decrease in serum IL-6 in the range of 0.39–0.45 pg/mL [31]. All *p*-values < 0.05 were considered statistically significant and data were analyzed using SAS/STAT software (Version 9.4; SAS Institute Inc., Cary, NC, USA).

## 3. Results

**Figure 1:**
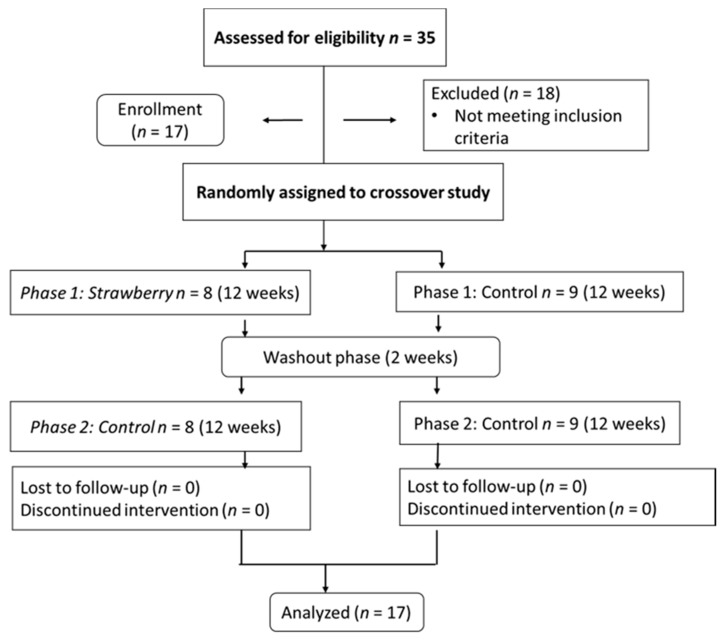
Study design.

Among the 35 participants who were screened, 17 qualified and completed the 26-week study (Figure 1). The baseline characteristics of these participants are shown in Table 2. There were no drop-outs in the study. Among the participants who completed the study, compliance was 100% for the strawberry group and 97% for the control group as assessed by mandatory thrice weekly visits, with the return of any unconsumed strawberry and control powder on the days the participants did not come to the clinic. No adverse events were reported in the study. As a measure of compliance, plasma ellagic acid was detectable in 17 participants in the strawberry phase (means ± SEMs (Standard error of means), 30.2 ± 3.6 ng/mL), whereas concentrations were not detectable at baseline, at the end of washout, and at the end of the control phase.

We examined associations of the selected biomarkers of inflammation and cartilage degradation with knee pain scores and HAQ-DI at baseline. As shown in Table 3, in a multivariable model at baseline, IL-6 was significantly associated with constant pain, and MMP-8 with intermittent knee pain (both *p* < 0.05). 

Among the selected inflammatory variables associated with knee OA measured in the study, serum IL-6 and IL-1β were significantly lower in the strawberry vs. control phase at week 12 (*p* < 0.05, Table 4), while no changes were noted in the serum hs-CRP and nitrite levels. Among the serum markers of cartilage degradation, MMP-3 was observed to be significantly lower in the strawberry vs. control phase at week 12 (*p* < 0.05, Table 4), while no significant changes were noted in MMP-8 between the two phases. As shown in Table 4, anthropometrics, blood pressure, glucose, HbA1c, lipid profiles, and liver and kidney function tests did not differ between the strawberry and control phases of the crossover study.

As shown in Table 5, the pain scores and HAQ-DI ratings were lower in the strawberry vs. control phase of the study. The knee pain scores measured as constant, intermittent, and total pain using ICOAP surveys were significantly lower following the strawberry vs. control phase at week 12 (*p* < 0.05, Table 5). No differences were noted in the VAS pain scores. Among the surveys related to general health and disability index, the HAQ-DI ratings were again significantly lower in the strawberry vs. control phase at week 12 (*p* < 0.05, Table 5). The VAS health scores were not affected by the strawberry treatment.

The dietary data did not reveal any significant differences in the mean intake of macro- and micro-nutrients throughout the study (Table 6). No crossover effects were detected on any of the outcome variables.

## 4. Discussion

To our knowledge, this is the first clinical study on the effects of dietary berries as a nutritional supplement on pain scores and key biomarkers of inflammation in obese adults with radiographic evidence of knee OA. Using a multi questionnaire approach, strawberry supplementation led to significant decreases in constant, intermittent, and total knee pain scores, and an improved disability index and overall health scores. Serum biomarkers of inflammation and cartilage degradation that have been associated with pain and dysfunction in knee OA, especially IL-6, IL-1β, and MMP-3, were also shown to be significantly lower in the strawberry-supplemented group. These findings support a role for foods high in bioactive compounds, such as strawberries, as an alternative or complementary treatment option in pain management that may also reduce surrogate markers of disease progression in knee OA.

Pain relief is one of the major targets of OA management. Symptoms of pain in OA are associated with inflammation and oxidative stress, cartilage degradation, and joint space narrowing [32,33,34]. It is therefore logical to propose that antioxidant supplements may be of benefit. Strawberries are naturally rich in antioxidant polyphenols, and thus were selected for our study [35]. Participants rated their pain in the range of mild to moderate intensity at baseline using the VAS as well as the ICOAP questionnaires. Other polyphenol containing supplements, such as curcumin [13,36], green tea [16], and herbal tea supplements [17], have also shown significant decreases in knee pain scores in participants with similar intensity of knee pain as in our study. However, none of these studies reported effects on systemic markers of inflammation and disease progression underlying knee OA. 

Together with previous reports, our findings support the analgesic effects of dietary polyphenols in adults with mild to moderate knee pain. Pain measurement in OA has been largely determined by questionnaires, such as those based on quality of life indicators. Meanwhile, physical examination and radiography have been used to stage the disease. We used the VAS, HAQ-DI, and ICOAP questionnaires: these have been widely employed to assess knee pain, quality of life, and disability in adults with OA [29,30]. The ICOAP questionnaire is endorsed by the Osteoarthritis Research Society International (OARSI). It has been validated in large multi-country studies, and correlates well with other commonly used methods, such as the Western Ontario and McMaster Universities’ Osteoarthritis Index (WOMAC) scores [28,37]. Based on our study findings, strawberries consistently improved pain scores as observed across all three sub-scales of ICOAP, evaluating constant, intermittent, and total pain; they also lowered HAQ-DI scores, reflecting functional improvement. We did not observe any difference in pain scores assessed by VAS survey in our participants. These differences could be explained by the visual expression of general pain intensity used in VAS scoring, when compared to the OA-specific magnitude of knee pain numerically rated by the ICOAP questionnaires. Based on the strengths and limitations of each assessment tool for pain, it is generally recommended to administer more than one questionnaire to capture the multi-dimensional aspects of adult pain. These findings merit follow up in larger trials to validate the findings.

Inflammation is believed to play a pivotal role in the pathophysiology of OA. Multiple cytokines and inflammatory molecules, especially CRP, IL-6, and IL-1β, and free radicals, such as nitric oxide, are implicated in the progression of OA [38,39]. Inflamed chondrocytes then produce MMPs, leading to cartilage degradation and progression of OA [40]. In experimental models of OA, green tea, curcumin, and some herbal supplements may reduce inflammatory molecules and MMPs [41,42], while few of the reported clinical studies have determined the effects of the dietary bioactive compounds on inflammatory mediators. In a 16-week study assessing the effects of a high-polyphenol rosmarinic acid tea on pain in participants with knee OA, serum CRP was reported only at baseline but not after the intervention [17]. Among the studies showing improvements in knee pain in OA following curcumin supplementation [13,43,44], only one study reported data on inflammatory markers, including IL-6; however, these did not differ between the intervention and placebo groups after six weeks’ treatment [43]. Thus, the study of inflammatory mediators is limited in previous reports of dietary supplements for OA.

In our study, 12 weeks of strawberry supplementation resulted in a significant decrease in IL-6, IL-1β, and MMP-3 in obese participants with knee OA, consistent with anti-inflammatory effects of dietary berries in OA management. These clinical observations are consistent with data showing that blueberry and raspberry extracts lower pain, inflammation, and edema, and articular destruction in experimental arthritis [21,22]. Metalloproteinases zinc-dependent enzymes (MMPs) play a key role in extracellular matrix remodeling and cartilage metabolism in knee OA. Among the various isoforms of MMPs, MMP-3 plays an important role in cartilage degradation, and has been shown to be responsive to therapeutic agents in patients with various stages of OA [12,45,46]. On the other hand, MMP-8 has been implicated in the degradation of already compromised cartilage matrix, and a few clinical studies have examined its response to therapeutic agents, and revealed conflicting results [45,47,48]. Thus, future studies must address the role of dietary polyphenolic compounds on a comprehensive panel of serum MMPs to identify clinically responsive biomarkers in OA management.

Obesity has been strongly correlated with knee OA, and consequently weight loss studies, especially the Intensive Diet and Exercise for Arthritis (IDEA) trial, have demonstrated significant decreases in IL-6 and improvements in knee pain and function following diet and exercise interventions in obese older adults [31]. Interestingly, the magnitude of the decrease in serum IL-6 in our study following the 12-week strawberry intervention was much larger than what was noted in the IDEA trial following an 18-month lifestyle intervention [31]. IL-6 is a key inflammatory molecule that accelerates articular degradation and OA progression, and higher levels of systemic IL-6 are a significant predictor of OA [49]. Furthermore, a reduction of IL-6 levels can significantly improve the metabolic syndrome, also considered a risk factor of OA [50]. The IDEA trial also reported a concomitant decrease in CRP in obese older adults undergoing 5% total weight and fat mass loss following a dietary and exercise intervention [51]. However, CRP was not significantly altered in the present study, and it may be that weight loss is essential to affect this marker of inflammation. Future studies must assess the combined effects of antioxidant bioactive compounds with weight loss in improving inflammatory profiles in knee OA.

Our study has limitations that affect the interpretation and generalizability of our findings. These include a small sample size, the absence of a dose–response design to assess effects at low vs. high dose of strawberries, and the absence of a non-OA control group. Participants had mild-to-moderate radiographic knee OA at baseline (Kellgren–Lawrence scores of 2.1) and mild-to-moderate knee pain. Whether patients with more severe knee OA (Kellgren–Lawrence score of 4) and higher levels of pain would benefit from strawberry intervention needs further investigation. We did not measure other biomarkers of OA pathology, such as those related to oxidative damage, or simultaneously measure these biomarkers in synovial fluid that would provide a more accurate determination of changes specific to the knee. Also, we did not assess the radiological outcomes at the end of the intervention. Also, being conducted in obese participants with mild-to-moderate symptoms of knee OA, our study findings may not be generalizable to the non-obese population or OA caused by sports injuries and other trauma, or to those needing pain relief after knee surgery.

The strengths of our study include a randomized, controlled cross-over study design, which accounts for most of the inter-individual variations in parallel arm studies. Also, based on the administration of a control powder that matched the freeze-dried strawberries in sensory qualities, we were able to keep the participants and study coordinators blinded to the identity of the test agents. In addition, we excluded participants who were taking supplements, such as fish oil and other herbal supplements for pain relief, as well as those participating in a weight loss program, and thus were able to exclude potential confounding by these factors.

## 5. Conclusions

In conclusion, our pilot study provides evidence on the role of strawberry bioactive compounds, as a rich source of polyphenols and nutrients, in improving pain and inflammation in obese adults with mild-to-moderate knee OA when compared to a control group. Given the economic burden of obesity and related conditions, including knee OA, our study suggests that simple dietary intervention, i.e., the addition of berries, may have a significant impact on pain, inflammation, and overall quality of life in obese adults with OA.

## Figures and Tables

**Table 1:** Composition of the freeze-dried strawberry and control powders ^1^.

Nutrients/Compounds	Strawberry Powder	Control Powder
Dose, g	50	50
Calories, kcal	160	172
Carbohydrates, g	35	38
Total polyphenols, mg GAE	1585	75
Total anthocyanins, mg cyanidin-3-glucoside equivalents	66	-
Ellagic acid, mg	220	-
Phytosterols, mg	50	-
Dietary fiber, g	8	5

^1^ Supplied by California Strawberry Commission (Watsonville, CA, USA); analysis conducted at the Robert M. Kerr Food and Agricultural Products Center at Oklahoma State University, polyphenol and anthocyanin analyzed by Brunswick Laboratories (Southborough, MA, USA); GAE: gallic acid equivalents.

**Table 2:** Baseline characteristics.

N	17
Age (years)	57 ± 7
Gender M/F	4/13
Anti-hypertensive medications (%)	65
Lipid lowering medications (%)	35
Oral hypoglycemic agents (%)	12
Vitamin supplement users (%)	65
Kellgren–Lawrence grade	2.1 ± 0.7

Values are in means ± SD for age (years); M: male; F: female.

**Table 3:** Baseline associations of knee pain scores and Health Assessment Questionnaire-Disability Index with selected markers of inflammation and cartilage degradation in obese adults with symptomatic knee osteoarthritis (linear regression coefficients) (*n* = 17).

Serum Variable	ICOAP (Constant Pain) %	ICOAP (Intermittent Pain) %	ICOAP (Total Pain) %	HAQ-DI
hs-CRP (mg/L)	1.9 ± 1.3	1.7 ± 1.4	1.8 ± 1.1	0.03 ± 0.04
*p*-value *	0.18	0.24	0.25	0.28
IL-6 (pg/mL)	5.7 ± 2.8	1.8 ± 2.9	3.6 ± 2.4	0.003 ± 0.08
*p*-value	**0.03**	0.23	0.37	0.42
IL-1β (pg/mL)	0.4 ± 0.3	0.04 ± 0.3	0.2 ± 0.1	0.003 ± 0.007
*p*-value	0.21	0.32	0.42	0.46
MMP-3 (ng/mL)	1.6 ± 1.7	1.9 ± 1.8	1.8 ± 1.5	0.03 ± 0.05
*p*-value	0.34	0.23	0.21	0.53
MMP-8 (ng/mL)	2.7 ± 3.4	6.9 ± 3.5	5.0 ± 2.9	0.01 ± 0.09
*p*-value	0.32	**0.006**	0.42	0.21

Values are estimate (beta) ± standard error (SE) obtained from a multiple linear regression model; For each serum variable, the model was adjusted for the remaining variables of inflammation and cartilage degradation in the model, as well as baseline age, body mass index (BMI), waist circumference, and energy intake as covariates; * *p* < 0.05 in bold; hs-CRP, high sensitivity C-reactive protein; IL-6: interleukin-6; IL-1β: interleukin-1β; MMP: matrix metalloproteinase; ICOAP: intermittent and constant osteoarthritis pain; HAQ-DI: health assessment questionnaire disability index.

**Table 4:** Anthropometrics and serum biochemical and inflammatory profiles following strawberry and control interventions in a 26-week crossover trial in obese adults with symptomatic knee osteoarthritis (*n* = 17/group).

Variables	Baseline	Strawberry (12-Week)	Washout (2-Week)	Control (12-Week)	*p*-Value *
BMI (kg/m^2^)	39.1 ± 1.5	39.3 ± 1.4	39.3 ± 1.5	39.3 ± 1.5	0.41
Body weight (lb)	246.4 ± 7.3	245.6 ± 7.4	245.1 ± 7.1	245.0 ± 7.2	0.32
Waist circumference (inches)	46.4 ± 1.1	46.5 ± 1.1	46.1 ± 1.0	46.8 ± 1.1	0.26
Systolic blood pressure (mm Hg)	125 ± 3.0	125 ± 2.0	126 ± 3.0	127 ± 2.0	0.37
Diastolic blood pressure (mm Hg)	80 ± 2.0	82 ± 1.0	81 ± 2.0	82 ± 1.0	0.41
Fasting glucose (mg/dL)	113.4 ± 5.6	118.9 ± 8.7	114.7 ± 4.5	112.3 ± 5.1	0.31
HbA1c (%)	5.9 ± 0.2	6.1 ± 0.1	5.99 ± 0.2	6.1 ± 0.2	0.88
Total Cholesterol (mg/dL)	189 ± 6.2	188 ± 7.6	182 ± 7.0	188 ± 8.7	0.86
LDL Cholesterol (mg/dL)	109 ± 6.3	108 ± 6.9	105 ± 6.8	105 ± 7.6	0.65
HDL Cholesterol (mg/dL)	51 ± 3.0	52 ± 2.9	50 ± 2.5	54 ± 3.3	0.12
Triglycerides (mg/dL)	129 ± 14.3	136 ± 16.1	128 ± 15.0	130 ± 12.9	0.54
ALT (U/L)	40.5 ± 2.8	38.6 ± 2.3	39.5 ± 2.1	40.6 ± 2.9	0.42
AST (U/L)	30.1 ± 2.6	28.9 ± 2.3	28.6 ± 1.7	31.4 ± 2.1	0.52
Creatinine (mg/dL)	0.75 ± 0.03	0.78 ± 0.03	0.75 ± 0.03	0.77 ± 0.02	0.42
BUN (mg/dL)	15.5 ± 0.9	14.5 ± 0.7	15.3 ± 1.0	14.5 ± 1.0	0.36
hs-CRP (mg/L)	5.7 ± 1.2	4.6 ± 0.9	5.4 ± 1.1	4.8 ± 0.8	0.74
IL-6 (pg/mL)	8.8 ± 0.4	3.4 ± 0.5	8.1 ± 0.9	8.7 ± 1.4	**0.006**
IL-1β (pg/mL)	18.6 ± 4.0	7.5 ± 0.7	16.3 ± 3.1	16.2 ± 1.2	**<0.0001**
MMP-3 (ng/mL)	6.9 ± 0.6	5.3 ± 0.5	7.1 ± 0.6	6.8 ± 0.5	**0.004**
MMP-8 (ng/mL)	1.8 ± 0.3	2.2 ± 0.3	2.4 ± 0.2	2.1 ± 0.2	0.26
Nitrite (μM)	6.4 ± 0.7	9.9 ± 2.2	6.4 ± 0.8	7.5 ± 0.8	0.23

Values are means ± SEMs (Standard error of means) obtained from a linear mixed-effects model with time as within-subject factor and intervention group as a between-subject factor; * Strawberry 12-week vs. Control 12-week adjusted for Baseline; *p* < 0.05 in bold; ALT: alanine aminotransferase; AST: aspartate aminotransferase; BMI: body mass index; BUN: blood urea nitrogen; hs-CRP, high sensitivity C-reactive protein; IL-6: interleukin-6; IL-1β: interleukin-1β; MMP: matrix metalloproteinase.

**Table 5:** Measures of knee pain and quality of life indicators following strawberry and control interventions in a 26-week crossover trial in obese adults with symptomatic knee osteoarthritis (*n* = 17/group).

Variables	Baseline	Strawberry (12-Week)	Washout (2-Week)	Control (12-Week)	*p*-Value *
HAQ-DI	0.6 ± 0.1	0.4 ± 0.1	0.5 ± 0.1	0.6 ± 0.1	**0.026**
VAS PAIN	1.4 ± 0.2	0.8 ± 0.1	1.1 ± 0.1	1.0 ± 0.2	0.17
VAS HEALTH	0.7 ± 0.1	1.0 ± 0.2	0.9 ± 0.2	0.7 ± 0.1	0.13
ICOAP (Constant pain) %	31.8 ± 3.5	13.8 ± 3.6	32.1 ± 3.7	24.2 ± 4.1	**0.01**
ICOAP (Intermittent pain) %	38.5 ± 3.4	24.3 ± 4.7	34.1 ± 2.2	34.6 ± 3.0	**0.02**
ICOAP (Total pain) %	35.4 ± 3.1	19.4 ± 3.7	33.2 ± 2.2	29.9 ± 3.0	**0.007**

Values are means ± SEMs obtained from a linear mixed-effects model with time as within-subject factor and intervention group as a between-subject factor; * Strawberry 12-week vs. Control 12-week adjusted for Baseline; *p* < 0.05 in bold; ICOAP: intermittent and constant osteoarthritis pain; HAQ-DI: health assessment questionnaire disability index; VAS: visual analog scale.

**Table 6:** Dietary nutrient intakes in a 26-week crossover trial in obese adults with symptomatic knee osteoarthritis (*n* = 17/group).

Nutrients	Baseline	Strawberry (12-Week)	Washout (2-Week)	Control (12-Week)	*p*-Value *
Calories (kcal)	2026 ± 183	2604 ± 441	2004 ± 184	1901 ± 203	0.12
Carbohydrates (g)	215 ± 19	320 ± 56	216 ± 20	228 ± 29	0.21
Fats (g)	90 ± 12	100 ± 22	89 ± 12	78 ± 9	0.31
Proteins (g)	92 ± 11	107 ± 19	96 ± 10	76 ± 7	0.43
Saturated fats (g)	30 ± 4	32 ± 6	28 ± 3	26 ± 3	0.31
MUFA (g)	17 ± 4	22 ± 6	17 ± 4	13 ± 3	0.18
PUFA (g)	8 ± 2	12 ± 3	7 ± 1.5	6 ± 2	0.11
Fiber (g)	18 ± 2	23 ± 3	19 ± 2	20 ± 3	0.43
Vitamin C (mg)	45 ± 9	81 ± 17	42 ± 9	75 ± 28	0.77
Vitamin E (mg)	5 ± 2	7 ± 2	4 ± 1	5 ± 2	0.34
Beta-carotene (μg)	590 ± 303	890 ± 203	580 ± 311	828 ± 400	0.63

Values are means ± SEMs obtained from a linear mixed-effects model with time as within-subject factor and intervention group as a between-subject factor; * Strawberry 12-week vs. Control 12-week adjusted for Baseline; MUFA: monounsaturated fatty acids; PUFA: polyunsaturated fatty acids.
